# Dual Regulatory Role of Chromatin Remodeler ISW1 in Coordinating Cellulase and Secondary Metabolite Biosynthesis in Trichoderma reesei

**DOI:** 10.1128/mbio.03456-21

**Published:** 2022-02-08

**Authors:** Yanli Cao, Renfei Yang, Fanglin Zheng, Xiangfeng Meng, Weixin Zhang, Weifeng Liu

**Affiliations:** a State Key Laboratory of Microbial Technology, Shandong University, Qingdao, People’s Republic of China; University of Georgia

**Keywords:** *Trichoderma reesei*, ISW1, cellulase, sorbicillinoid, gene regulation

## Abstract

The saprophytic filamentous fungus Trichoderma reesei represents one of the most prolific cellulase producers isolated from nature. *T. reesei* also produces a typical yellow pigment identified as sorbicillinoids during cultivation. Here, we identified an evolutionarily conserved histone remodeling factor, ISW1, in *T. reesei* that simultaneously participates in regulating cellulase and the yellow pigment biosynthesis. *Trisw1* deletion almost abolished vegetable growth, asexual spore formation, and cellulase gene expression. However, its absence significantly enhanced the production of the yellow pigment. The observed dual regulatory role of TrISW1 was dependent on its ATPase activity. We demonstrated that *Trisw1* disruption elevated the transcription of *ypr1* coding for the transcriptional activator of *sor* genes encoding the polyketide synthases catalyzing the biosynthesis of sorbicillinoids but compromised that of *xyr1* encoding the key transcriptional activator of cellulase genes. Discrete *T. reesei* homologous ISW1 accessory factors were also found to exert differential effects on the expression of these two types of genes. Further analyses showed that TrISW1 was recruited to cellulase gene promoters, and its absence interfered with loss of histone H4 at the *cbh1* and *eg1* promoters upon cellulose induction. To the contrary, *Trisw1* deletion facilitated loss of H4 at the *sor* locus. These data indicate that TrISW1 represents an important chromatin remodeler with a dual role in coordinating the cellulolytic response and biosynthesis of the major secondary metabolite in *T*. *reesei*.

## INTRODUCTION

Dynamic modulation of chromatin structure and fine-tuning gene expression are intimately related key events in the control of eukaryotic gene expression. For transcriptional activation in eukaryotic cells, chromatin structure must be actively remodeled at gene promoters to counteract the repressive effect elicited by chromosomes packaged into nucleosomes and even higher-order structures. Two major classes of chromatin-modulating complexes, histone modifying enzymes and ATP-dependent chromatin remodelers, play key roles in these dynamic changes in the chromatin status (1, 2). Instead of posttranslationally adding or removing specific chemical groups on histones, chromatin remodeling complexes disrupt histone-DNA contacts, resulting in sliding of or even evicting the histone octamer on DNA using ATP hydrolysis by the catalytic subunit. By doing so, the underlying DNA sequence otherwise packaged into chromatin would be accessible to specific *trans*-acting factors and the general transcriptional machinery for successful gene transcription (3–5). Besides the founding member SWI/SNF (switch/sucrose nonfermentable) complex, three other subfamilies of ATP-dependent chromatin remodelers, ISWI, CHD1, and INO80, have been also described (6).

The ISWI (imitation SWI/SNF) subfamily of ATP-dependent chromatin remodelers was first identified using *in vitro* assays for nucleosome-remodeling activities in *Drosophila* embryo extracts (7). Whereas ISWI is a highly conserved member of the SWI2/SNF2 family of ATPases, it is distinguished from the other SNF2/SWI2-related subfamilies by the presence of HAND-SANT-SLIDE domains at its C-terminal half (8, 9), which have been proven to be putative DNA and nucleosome binding domains that play an important role in both transcriptional activation and repression (8–11). It has been shown that ISWI usually interacts with multiple accessory subunits to form distinct complexes, and these various accessory subunits are required to regulate the localization and catalytic activity of ISWI complexes (12–18). Therefore, the *Drosophila* ISWI constitutes the catalytic subunit of three distinct chromatin-remodeling complexes—ACF (ATP-dependent chromatin assembly and remodeling factor), NURF (nucleosome remodeling factor), and CHRAC (chromatin accessibility complex) (19–21). Similarly, in budding yeast, two closely related ISWI proteins, ISW1p and ISW2p, can, respectively, assemble with one to three accessory subunits to form three to seven different complexes (12, 22, 23). ISWI complexes have thus been the most diverse chromatin remodelers in terms of form and function.

One of the first indications that the ISWI complex promotes gene activation in a chromatin context comes from the observation that NURF directly facilitates GAL4-mediated transcription from chromatin templates *in vitro* (24). The role of NURF in transcriptional activation is further supported by the fact that lack of the largest accessory subunit of the NURF complex, NURF301, in *Drosophila*, reduces *hsp70* and *hsp26* gene transcription (25). A variety of sequence-specific transcription factors have been shown to physically interact with NURF301 *in vitro*, providing a potential mechanism for recruiting NURF to specific genes (26). On the other hand, there have also been results implicating ISWI in transcriptional repression. Therefore, while yeast ISW1p has been shown to either repress or promote transcription depending on which protein it interacts with (12, 27), ISW2p mainly exerts a repressive effect by positioning nucleosomes that obstruct transcription (28, 29). This is best exemplified by the transcriptional repression of early meiotic genes during mitotic growth wherein the suppressor UME6P recruits the ISW2 complex to fulfill the inhibition (28).

The filamentous fungus Trichoderma reesei is well known for its outstanding cellulase-secreting capability and also represents an important model fungus for studying the induced eukaryotic gene expression. Among various transcriptional factors that have been identified, XYR1 has been considered a master transcriptional factor controlling the induced expression of almost all cellulase and hemicellulose genes (30). Apart from these, changes in the chromatin status of cellulase genes have also been observed in context to the applied conditions (repressing/inducing) (31, 32). Our previous work has revealed that XYR1 recruits the SWI/SNF complex to remodel chromatin at cellulase gene promoters, thereby activating cellulase gene expression to initiate the cellulolytic response in *T*. *reesei* (33).

During cultivation, *T. reesei* also produces a typical yellow pigment that has been identified as sorbicillinoids belonging to hexaketide metabolites (34, 35). Sorbicillinoids are synthesized by a gene cluster including two polyketide synthase-encoding genes, *sor1* and *sor2*. Two transcription factors (YPR1 and YPR2) that are encoded in the same cluster have been shown to regulate the expression of the *sor* genes (35). While YPR1 is considered to be the transcriptional activator for the expression of most genes within the cluster, YPR2 acts as a repressor that negatively regulates the expression of the *ypr1* and *sor* genes (35). Although the exact mechanism by which YPR1 and YPR2 regulate the *sor* expression is not clear at present, it has been found that excessive formation of the yellow pigment dramatically interferes with the induced cellulase gene expression (36).

In this study, we found that disruption of *Trisw1* encoding the ATPase subunit of ISWI in *T. reesei* abolished the induced production of cellulases but greatly enhanced the yellow pigment formation. Evidence was provided that TrISW1 differentially affected the expression of the two activators responsible for cellulase gene and *sor* gene transcription and that this function depended on its ATPase activity. We also analyzed the role of various homologous ISWI accessory subunits in regulating the *sor* and cellulase gene expression and provided genetic evidence that distinct ISWI complexes may function at these target genes. TrISW1 was further shown to be recruited to cellulase gene promoters in the presence of cellulose and polyketide synthase (PKS) genes promoters regardless of the kind of carbon source. Moreover, TrISW1 displayed opposing effects on the occupancy of histone H4 at cellulase gene and PKS-related gene promoters.

## RESULTS

### Disruption of *Trisw1* compromised *T. reesei* vegetative growth and conidiation but significantly enhanced the production of the yellow pigment.

**FIG 1:**
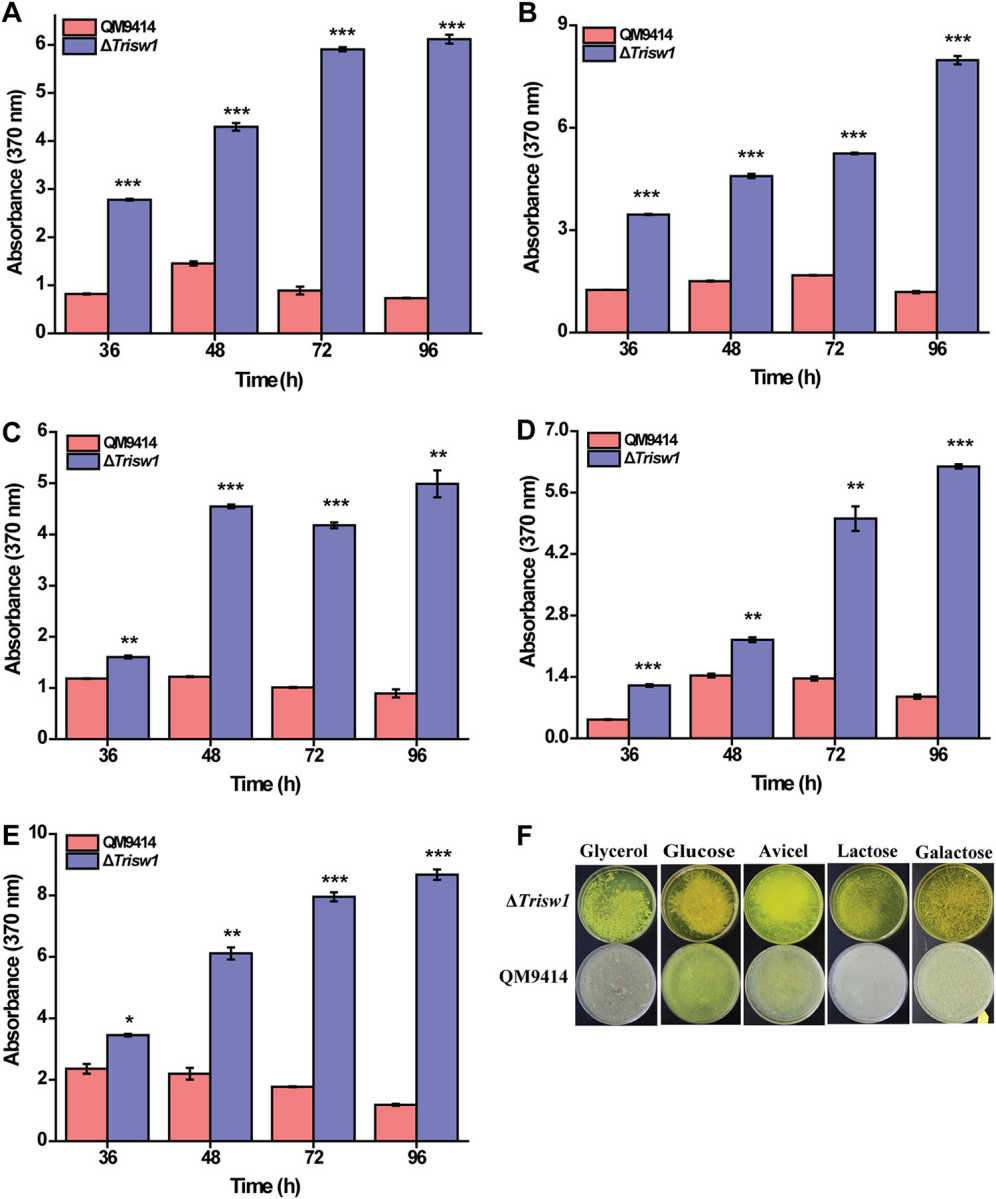
Sorbicillinoid production in *T. reesei* QM9414 and Δ*Trisw1* grown on different carbon sources. (A to E) The culture supernatant of the QM9414 and Δ*Trisw1* strains cultivated in MA liquid medium containing glycerol (A), glucose (B), Avicel (C), lactose (D), or galactose (E) at a final concentration of 1% (wt/vol) was assayed for sorbicillinoids by measuring the absorbance at 370 nm at the indicated time points. (F) The apparent color of the corresponding culture supernatant at 96 h. Significant differences (*t* test *, *P* < 0.05; **, *P* < 0.01; ***, *P* < 0.001) were observed in the sorbicillinoid production between QM9414 and Δ*Trisw1* for the indicated time points.

Four distinct chromatin remodeling complexes, SWI/SNF, ISWI, INO80, and CHD1, have been described in eukaryotes (6, 37). Besides SWI/SNF, *in silica* analysis showed that the *T. reesei* genome also harbors the homologous catalytic subunit of three other complexes (Fig. S1). To investigate the potential role of these putative chromatin remodelers in cellulase gene expression, we individually deleted the identified ATPase subunit containing the conserved catalytic core domain. The results showed that disruption of *Trino80* (Tr_50539) or *Trchd1* (Tr_58928) hardly affected the induced cellulase production (Fig. S2). Unlike TrINO80 and TrCHD1, deletion of *Trisw1* (Tr_57608) led to severely reduced vegetative growth and conidiation on agar plates (Fig. S3A). Growth of the Δ*Trisw1* strain was also compromised when cultured in liquid MA medium in the presence of glucose, glycerol, lactose, or Avicel (Fig. S3B to E). These results indicated that TrISW1 plays an important role in vegetative growth and asexual spore formation in *T. reesei*. Of note, Δ*Trisw1* secreted a much larger amount of yellow pigment than the parent strain (Fig. 1).

10.1128/mbio.03456-21.3 FIG S1 Schematic diagram of TrISW1, TrCHD1, TrINO80, and their counterparts in Saccharomyces cerevisiae. The conserved domains of the homologous catalytic subunit of ISWI, INO80, and CHD1 complexes in *T. reesei* and S. cerevisiae as well as the identities are indicated. Download FIG S1, TIF file, 0.6 MB. Copyright © 2022 Cao et al. 2022 Cao et al. https://creativecommons.org/licenses/by/4.0/ This content is distributed under the terms of the Creative Commons Attribution 4.0 International license.

10.1128/mbio.03456-21.4 FIG S2 Effect of Δ*Trchd1* or Δ*Trino80* deletion on growth, cellulase gene expression, and the sorbicillinoid production. (A) Growth of QM9414, Δ*Trchd1*, and Δ*Trino80* strains on MA agar plates containing 1% glucose as the carbon source. (B and C) Extracellular *p*NPC hydrolytic activities of QM9414 and three independent transformants of Δ*Trchd1* (B) or two independent transformants of Δ*Trino80* (C) cultured on 1% (wt/vol) Avicel were determined for the indicated time periods. No significant difference (*t*-test *P* > 0.05 [n.s.]) was observed for *p*NPC activities between QM9414 and Δ*Trchd1* or between QM9414 and Δ*Trino80* strains at the indicted time points. (D) Biomass accumulation of the QM9414, Δ*Trchd1*, and Δ*Trino80* strains in MA liquid culture with glucose. (E) The sorbicillinoid production was assayed by measuring the absorbance at 370 nm at the indicated time points. Deletion of Δ*Trchd1* or Δ*Trino80* has no significant effect on growth and sorbicillinoid production. Download FIG S2, TIF file, 1.2 MB. Copyright © 2022 Cao et al. 2022 Cao et al. https://creativecommons.org/licenses/by/4.0/ This content is distributed under the terms of the Creative Commons Attribution 4.0 International license.

10.1128/mbio.03456-21.5 FIG S3 The effect of *Trisw1* deletion on the vegetative growth and conidiation of *T. reesei*. (A) Growth of the QM9414 and Δ*Trisw1* strains on plates with various carbon sources at a final concentration of 1% (wt/vol) at 30°C for 3 days and conidia formation on malt extract for 5 days. (B to E). Biomass accumulation of QM9414 and Δ*Trisw1* strains in MA liquid culture with glucose (B), glycerol (C), lactose (D), or Avicel (E) at a final concentration of 1% (wt/vol), respectively. Significant differences (*t*-test *, *P < *0.05; **, *P < *0.01; ***, *P < *0.001) were observed in the growth between QM9414 and Δ*Trisw1* for the indicated time points. Download FIG S3, TIF file, 2.8 MB. Copyright © 2022 Cao et al. 2022 Cao et al. https://creativecommons.org/licenses/by/4.0/ This content is distributed under the terms of the Creative Commons Attribution 4.0 International license.

Liquid chromatography-mass spectrometry (LC-MS) analysis of the extracted culture supernatant of the Δ*Trisw1* strain revealed that four known sorbicillinoid-related compounds were produced—sorbicillin, sorbicillinol, bisorbicillinol, and bisvertinolone (Table S1 and Fig. S4). Two compounds with unknown structures were also detected. In agreement with previous reports (35, 38), sorbicillinol was the most abundant product and is considered to be the building block for other sorbicillinoids.

10.1128/mbio.03456-21.1 TABLE S1 LC-MS results of the extracted culture broth of Δ*Trisw1* grown on galactose for 96 h. Download Table S1, DOCX file, 0.01 MB. Copyright © 2022 Cao et al. 2022 Cao et al. https://creativecommons.org/licenses/by/4.0/ This content is distributed under the terms of the Creative Commons Attribution 4.0 International license.

10.1128/mbio.03456-21.6 FIG S4 HPLC analyses of the organic phase of the fermentation liquid from QM9414 and Δ*Trisw1* cultured on galactose for 96 h. A 10-μL extracted sample filtered using a 0.22-μm aperture filter was injected into a C_18_ column at a flow rate of 1 mL/min using H_2_O plus 0.1% formic acid and acetonitrile plus 0.1% formic acid as the mobile phase. Download FIG S4, TIF file, 0.3 MB. Copyright © 2022 Cao et al. 2022 Cao et al. https://creativecommons.org/licenses/by/4.0/ This content is distributed under the terms of the Creative Commons Attribution 4.0 International license.

### TrISW1 is required for the induced cellulase gene expression but represses sorbicillinoid-related gene expression.

**FIG 2:**
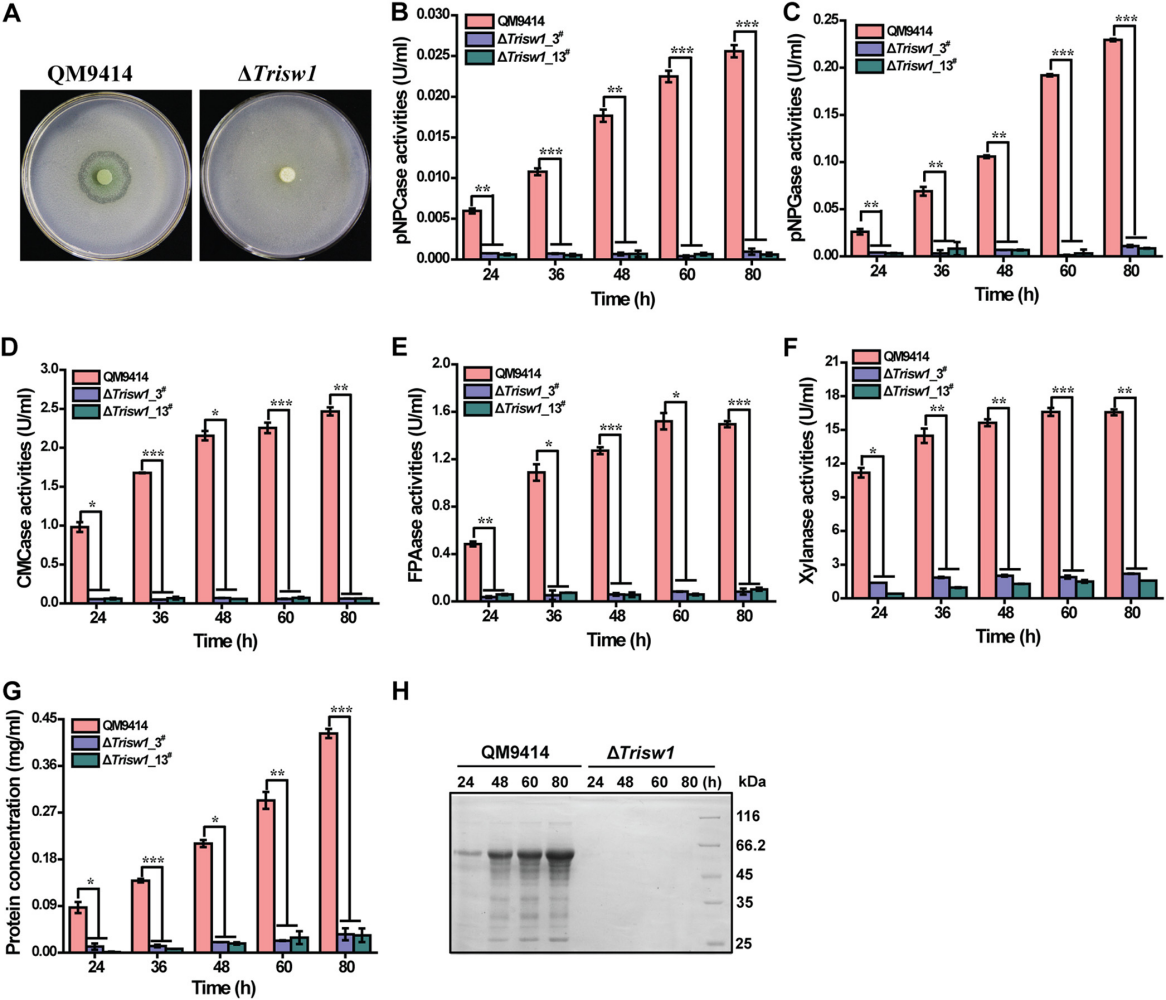
*Trisw1* deletion abolished (hemi)cellulase gene expression. (A) Hydrolytic zone formation by the QM9414 and Δ*Trisw1* strains on MA agar plates covered with a layer of 0.4% (wt/vol) ground Avicel. (B to G) Extracellular *p*NPC (B), *p*NPG (C), CMCase (D), filter paper activities (FPA) (E), xylanase activities (F), and protein concentration (G) of the culture supernatant of the parent strain QM9414 and two independent transformants of Δ*Trisw1* cultured on 1% (wt/vol) Avicel for the indicated time periods. (H) Culture supernatant of QM9414 and Δ*Trisw1* on 1% (wt/vol) Avicel was analyzed by SDS-PAGE and Coomassie brilliant blue staining. Significant differences (*t* test *, *P* < 0.05; **, *P* < 0.01; ***, *P* < 0.001) were observed for the extracellular activities between QM9414 and two independent transformants of Δ*Trisw1* for the indicated time points after induction.

**FIG 3:**
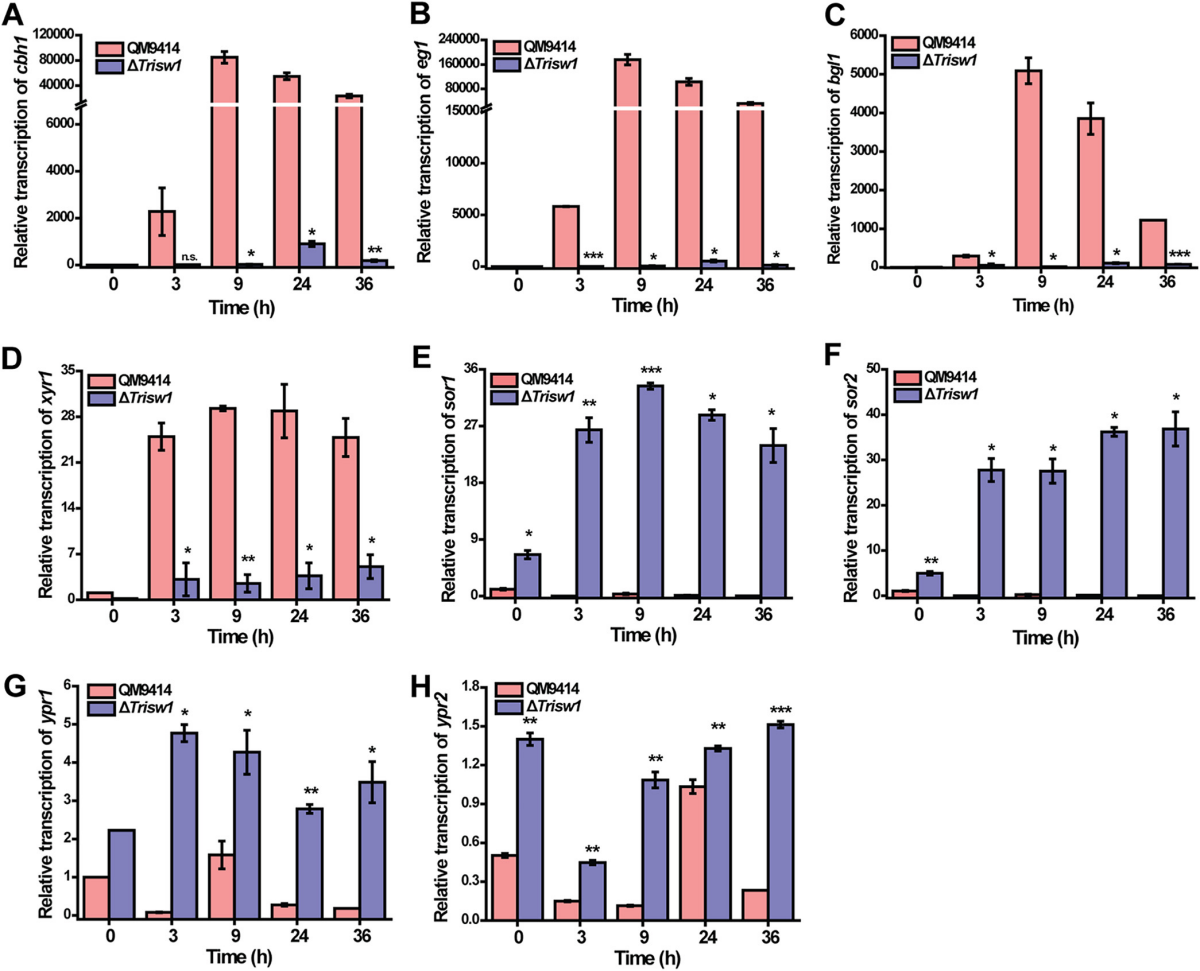
Deletion of *Trisw1* abolished the transcription of cellulase genes but significantly enhanced the transcription of sorbicillinoid biosynthetic genes. (A to H) Transcription of *cbh1* (A), *eg1* (B), *bgl1 (*C), *xyr1* (D), *sor1* (E), *sor2* (F), *ypr1* (G), and *ypr2* (H) was analyzed by quantitative RT-PCR with induction on 1% (wt/vol) Avicel for the indicated time points. The expression level of the actin gene was used as an endogenous control for all samples. Significant differences (*t* test *, *P* < 0.05; **, *P* < 0.01; ***, *P* < 0.001) were observed for the transcription of all detected genes between QM9414 and Δ*Trisw1* for the indicated time points.

To investigate the role of TrISW1 in cellulase gene expression, Δ*Trisw1* and the control strain QM9414 were individually inoculated on minimal medium (MM) agar plates covered with a top layer of 0.4% (wt/vol) ground Avicel. After incubation at 30°C for 6 days, QM9414 but not the Δ*Trisw1* colony produced an apparent hydrolytic halo (Fig. 2A), indicating that *Trisw1* deletion impaired cellulase biosynthesis. To further verify the effect of the absence of TrISW1 on cellulase production, the extracellular hydrolytic activities, including *p*NPCase, *p*NPGase, CMCase, FPAase, xylanase, and extracellular protein concentrations, were determined using Δ*Trisw1* and QM9414 culture supernatant on cellulose. Compared to QM9414, the absence of TrISW1 abolished cellulase and xylanase production (Fig. 2B to G). SDS-PAGE analysis confirmed that extracellular secreted proteins were hardly detected in Δ*Trisw1* (Fig. 2H). Further quantitative reverse transcription PCR (RT-PCR) analyses demonstrated that the relative transcriptional expression of cellulase genes *cbh1*, *eg1*, and *bgl1* and that of *xyr1* were almost eliminated in the Δ*Trisw1* strain (Fig. 3A to D). Contrary to the induction defect in cellulase genes, the transcription of *sor1*, *sor2*, *ypr1*, and *ypr2* was significantly enhanced in Δ*Trisw1* compared with QM9414 at all indicated time points (Fig. 3E to H).

**FIG 4:**
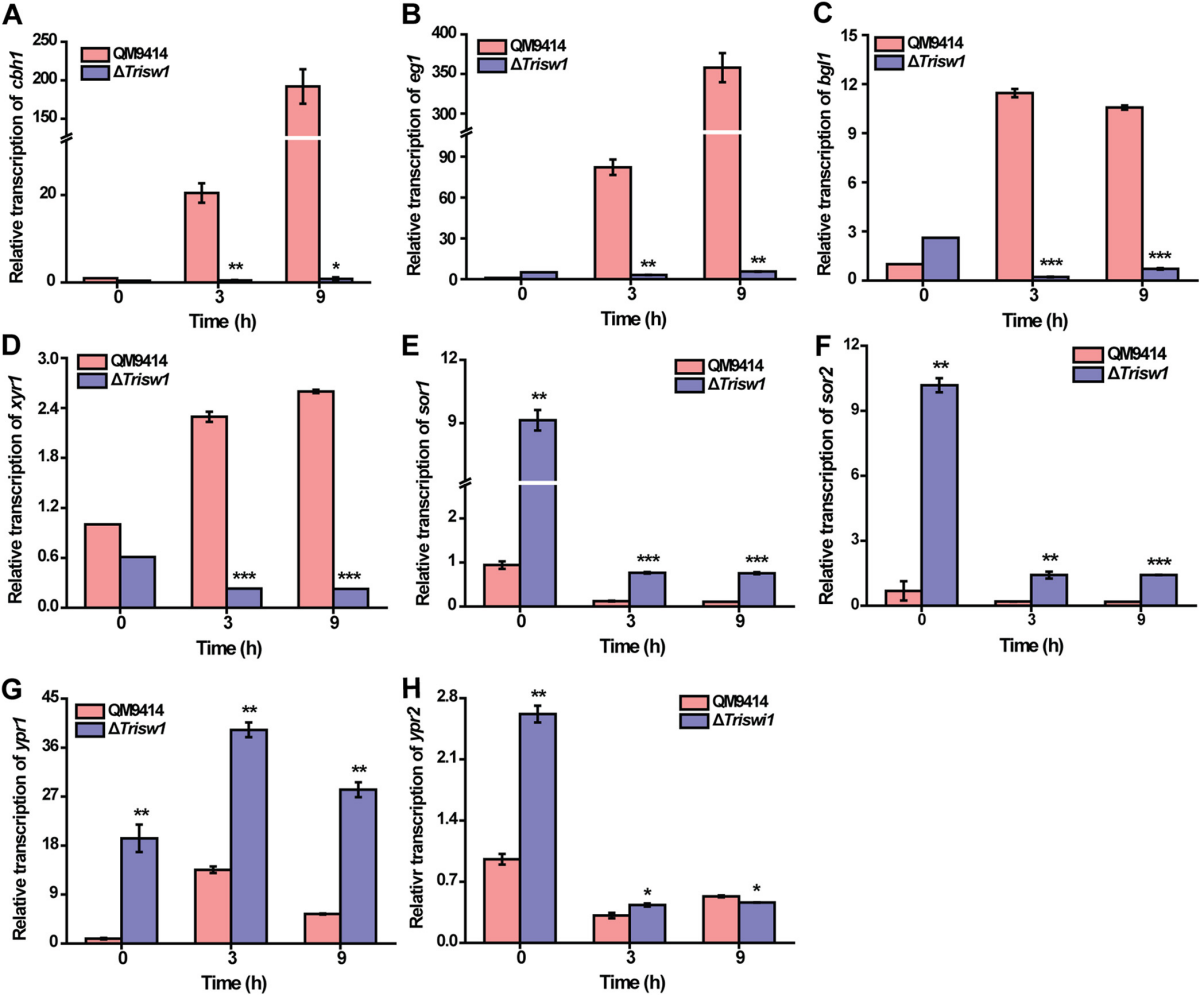
*Trisw1* deletion abolished the induced expression of cellulase genes but significantly enhanced the transcription of *sor*-related genes in a resting-cell system. (A to H) Quantitative RT-PCR analyses of the relative transcription of *cbh1* (A), *eg1* (B), *bgl1* (C), *xyr1* (D), *sor1* (E), *sor2* (F), *ypr1* (G), and *ypr2* (H) in the QM9414 and Δ*Trisw1* strains cultured in a resting-cell inducing system. The expression level of the actin gene was used as an endogenous control in all samples. Significant differences (*t* test *, *P* < 0.05; **, *P* < 0.01; ***, *P* < 0.001) were observed for the transcription of all detected genes between QM9414 and Δ*Trisw1* for the indicated time points.

To exclude the possibility that the observed induction deficiency was resultant from the growth defect of Δ*Trisw1*, the effect of *Trisw1* deletion on cellulase and *xyr1* gene transcription was analyzed in a resting system without any nitrogen or phosphate source. As shown in Fig. 4A to D, *Trisw1* deletion indeed severely compromised the rapid induction of cellulase and *xyr1* gene transcription. The increase in *sor* transcription was again observed in the same resting system used for cellulase gene analysis (Fig. 4E to H). Altogether, the data indicate that *Trisw1* plays an important role in mediating the induced expression of (hemi)cellulases, while it exerts a repressive effect on the expression of the *sor* cluster responsible for the biosynthesis of sorbicillinoids.

### TrISW1 is a nuclear protein, and its regulatory role depends on its ATPase activity.

**FIG 5:**
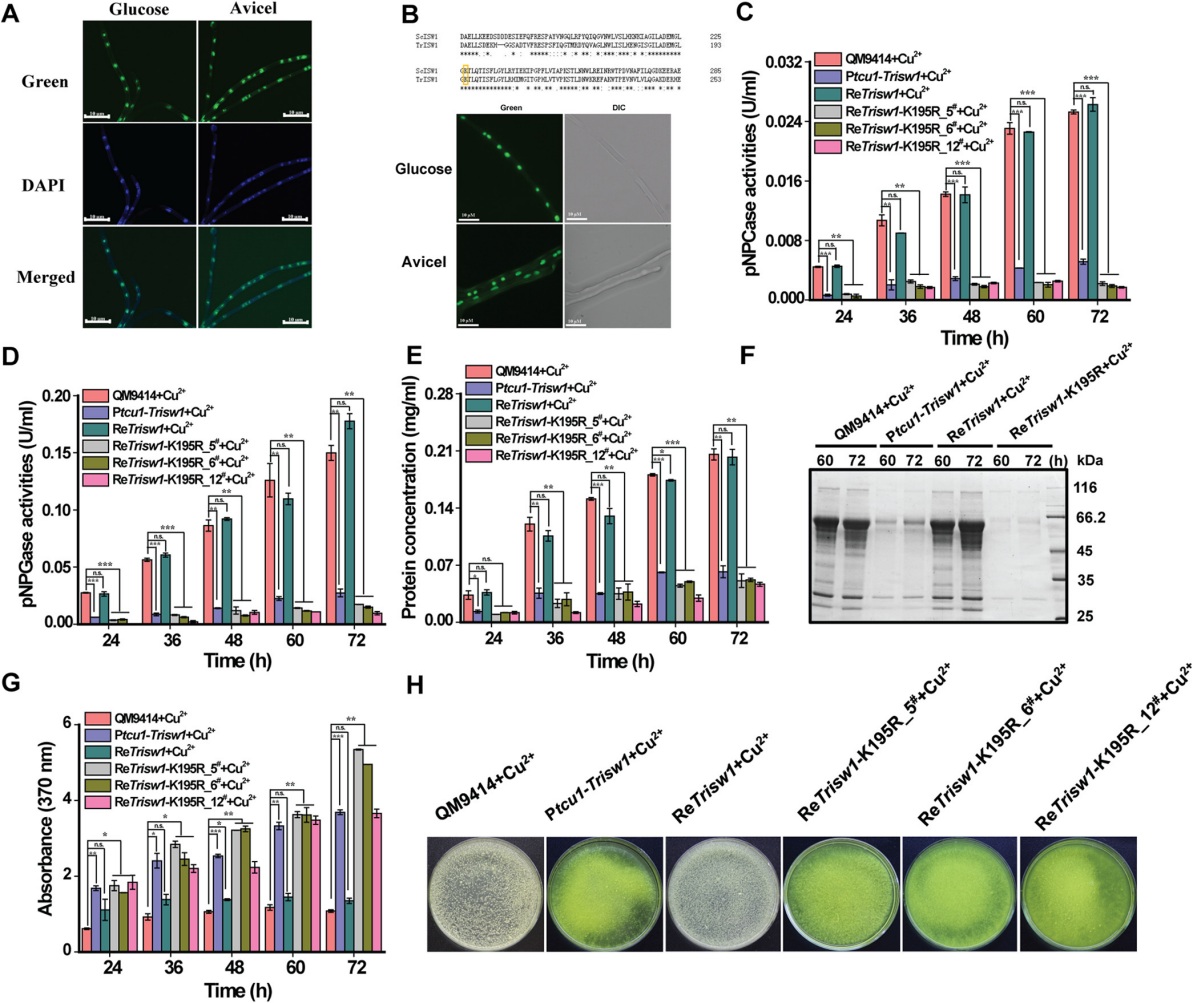
The ATPase activity of TrISW1 was critical for its regulatory role in *T. reesei*. (A) Subcellular localization of TrISW1 tagged with GFP at the N terminus. The conidia of the GFP-*Trisw1* strain were germinated on MA medium containing 1% (wt/vol) glucose for 16 h or 1% (wt/vol) Avicel for 24 h. The fluorescence was directly examined with a Nikon Eclipse 80i fluorescence microscope, and the nuclei were visualized by DAPI staining (blue). The results shown represent one of at least two independent experiments. (B) Amino acid sequence alignment of the ATPase domain of ScISW1 and TrISW1. The location of the conserved lysine residue mutated to arginine in this study is highlighted in a yellow circle. The GFP-TrISW1-K195R conidia were germinated on MA medium containing 1% (wt/vol) glucose for 16 h or 1% (wt/vol) Avicel for 24 h, and the fluorescence was directly examined with a Nikon Eclipse 80i fluorescence microscope. (C to E) Extracellular hydrolytic activities of *p*NPCase (C), *p*NPGase (D), and protein concentration (E) of the QM9414, P*tcu1*-*Trisw1*, and Re*Trisw1* strains as well as three independent transformants of Re*Trisw1*-K195R cultured on 1% (wt/vol) Avicel supplied with copper. (F) Culture supernatant of QM9414, P*tcu1*-*Trisw1*, Re*isw1*, and three independent transformants of Re*Trisw1*-K195R was analyzed by SDS-PAGE and Coomassie brilliant blue staining. (G) Sorbicillinoid production in the culture supernatant of the above-listed strains on 1% (wt/vol) Avicel was assayed by measuring the absorbance at 370 nm at the indicated time points (upper panel). (H) The apparent color of the corresponding culture supernatant at 72 h. Significant differences (*t* test *, *P* < 0.05; **, *P* < 0.01; ***, *P* < 0.001) were observed for extracellular activities and sorbicillinoid production between QM9414 and P*tcu1*-*Trisw1* or QM9414 and three independent transformants of Re*Trisw1*-K195R for the indicated time points with copper. No significant differences (*t* test *P* > 0.05 [n.s.]) were observed in the phenotypes between P*tcu1*-*Trisw1* and Re*isw1* for the indicated time points.

To determine the subcellular localization of TrISW1 in *T. reesei*, TrISW1 fused with an enhanced green fluorescent protein (EGFP) at its N terminus was expressed under the *tcu1* promoter in QM9414. The expression of this GFP-tagged TrISW1 did not interfere with its normal function (data not shown). As shown in Fig. 5A, TrISW1 was mainly localized in the nucleus. ISW1 protein and its catalytic core are evolutionarily conserved across species. It has been shown that the K227A mutant of yeast ISW1 leads to the loss of its ATPase activity. The mutant thus cannot restore the viability of the *yISW1* deletion strain at an elevated growth temperature (39). To investigate whether the ATPase activity of TrISW1 is directly involved in the regulation of cellulase and sorbicillinoid biosynthesis, we first applied the promoter substitution strategy to achieve one-step replacement of the endogenous *Trisw1* promoter with the P*tcu1* promoter (60), considering that the growth and conidiation defect of Δ*Trisw1* made it difficult to complement the deletion strain. When *Trisw1* was repressed, the resultant P*tcu1*-*Trisw1* displayed a phenotype that resembled the Δ*Trisw1* deletion strain (as shown below). We then introduced wild-type TrISW1 and TrISW1-K195R mutant (corresponding to yISW1 K227R) into P*tcu1*-*Trisw1*, respectively (Fig. 5B). The target mutation exerted hardly any effect on the expression of the introduced mutant TrISW1. While cellulase biosynthesis and yellow pigment production were corrected with WT TrISW1, TrISW1-K195R failed to bring back the cellulase and yellow pigment formation to a wild-type level (Fig. 5C to H). These results suggest that the ATPase activity of TrISW1 was critical for its regulating the cellulase and *sor* gene expression, most probably by remodeling nucleosomes positioned at relevant genes.

### Differential roles of ISWI accessory subunits in cellulase and sorbicillinoid-related gene expression.

**FIG 6:**
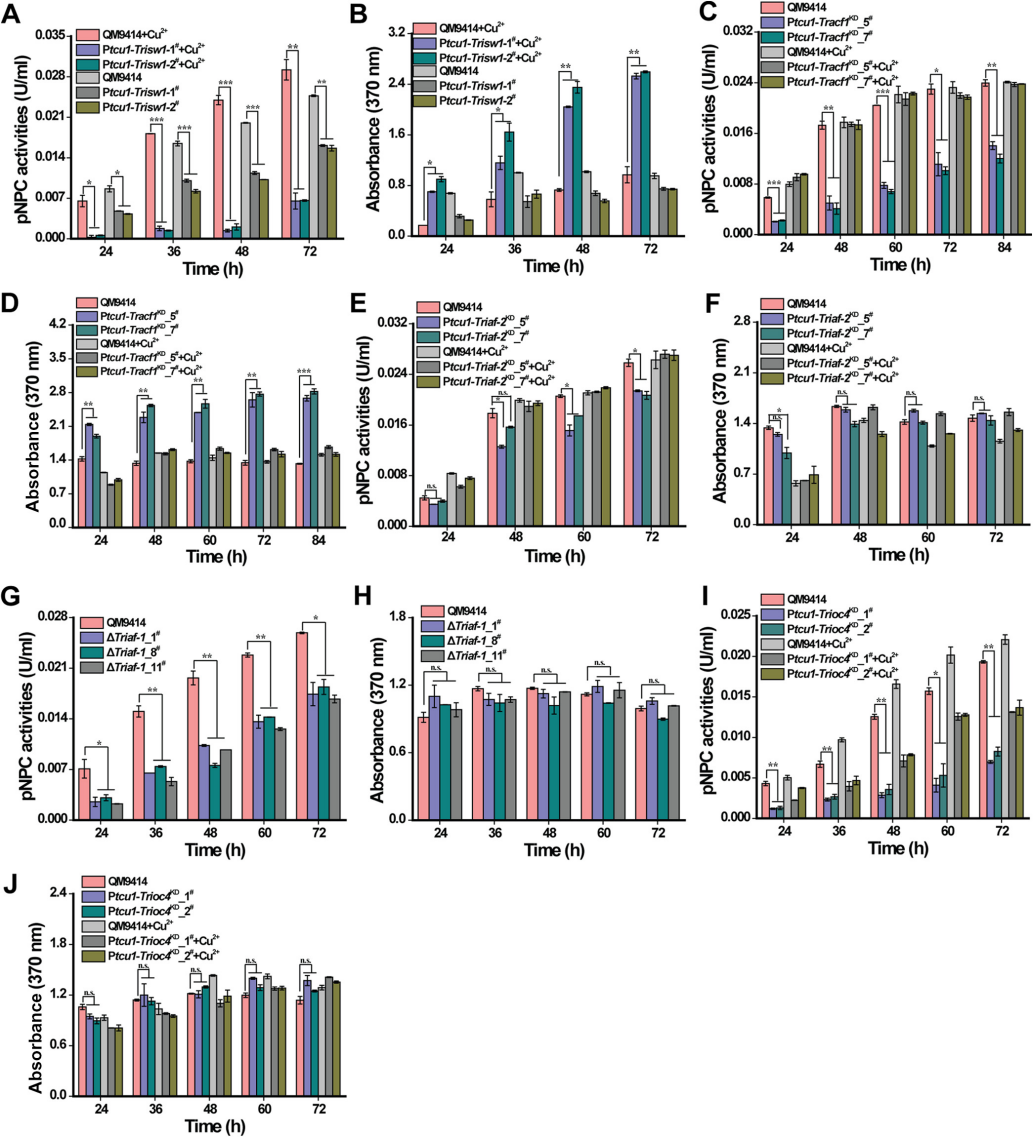
ISWI accessory subunits play differential roles in cellulase and sorbicillinoid-related gene expression. (A, C, E, G, and I) Extracellular *p*NPC of the culture supernatant from QM9414 and two independent transformants of P*tcu1*-*Trisw1*, P*tcu1*-*Tracf-1*^KD^, P*tcu1*-*Triaf-2* ^KD^, Δ*Triaf-1*, and P*tcu1*-*Trioc4*^KD^ cultured on 1% (wt/vol) Avicel for the indicated time periods with or without copper as indicated for controlling RNA interference. (B, D, F, H, and J) The sorbicillinoid production of the above-listed strains cultured on 1% (wt/vol) Avicel was determined by measuring the absorbance at 370 nm at the indicated time points. Significant differences (*t* test *, *P* < 0.05; **, *P* < 0.01; ***, *P* < 0.001) were observed for extracellular activities between QM9414 and Δ*Triaf-1*, P*tcu1*-*Tracf-1*^KD^, and P*tcu1*-*Trioc4*^KD^ without copper as well as between QM9414 and P*tcu1*-*Trisw1* with or without copper for the indicated time points. No significant difference (*t* test *P* > 0.05 [n.s.]) was observed for extracellular activities between QM9414 and P*tcu1*-*Triaf-2*^KD^ cultured without copper. Significant differences (*t* test *, *P* < 0.05; **, *P* < 0.01; ***, *P* < 0.001) were also observed for sorbicillinoid production between QM9414 and P*tcu1*-*Trisw1* with copper as well as between QM9414 and the P*tcu1*-*Tracf-1*^KD^ strain cultured without copper, but no significant difference (*t* test *P* > 0.05 [n.s.]) was observed between QM9414 and the Δ*Triaf-1*, P*tcu1*-*Trioc4*^KD^, and P*tcu1*-*Triaf-2* ^KD^ strains regarding sorbicillinoid production.

It has been shown that ISW homologs interact with various accessory proteins to form multiple complexes with distinct functions (15, 16, 18, 23, 27). Most recently, three ISW-containing complexes with five discrete accessory subunits have been identified in *Neurospora* (18). *In silico* analyses revealed that the *T. reesei* genome also contains predicted orthologs for most accessory components of ISW complexes (Table S2). Although the amino acid similarity between these *T. reesei* and yeast orthologs are relatively low (identity, <30%), significantly higher similarity can be found between *T. reesei* and Neurospora crassa counterparts (identity,50 to 60%), suggesting that ISW may also form multiple complexes in *T. reesei* through interactions with distinct accessory subunits. To determine if any of the identified individual homologous ISW accessory subunits participate in the observed differential gene regulatory roles of TrISW1, mutants with either gene deletion (Δ*Triaf-1*) or copper-controlled gene repression (P*tcu1*-*Trioc4*^KD^, P*tcu1*-*Tracf-1*^KD^, and P*tcu1*-*Triaf-2*^KD^, respectively) were constructed. P*tcu1*-*Trisw1* was also included for comparison. In contrast with the *Trisw1* shutdown mutant, growth of all accessory subunit mutants was largely unaffected when relevant target genes were deleted or repressed in the presence of glucose (Fig. S5). As shown in Fig. 6, while cellulase gene expression was severely compromised when the expression of *Trisw1* was repressed in the resultant P*tcu1*-*Trisw1* strain with addition of copper, the yellow pigment production was significantly increased (Fig. 6A and B and Fig. S6). *Trisw1* overexpression without copper also seemed to interfere with cellulase induction, but the pigment remained comparable to that of the parental strain (Fig. S6). Similar to *Trisw1*, *Tracf-1* repression in the absence of copper led to significantly compromised cellulase expression but increased production of sorbicillinoids (Fig. 6C and D). Unlike *Trisw1* and *Tracf-1*, repressing *Triaf-2* without copper hardly affected either cellulase or sorbicillinoid production (Fig. 6E and F). Interestingly, whereas disruption of *Triaf-1* or *Trioc4* repression reduced the induced cellulase production, neither had any effect on sorbicillinoid production (Fig. 6G to J). Taken together, these data demonstrate that *T. reesei* discrete accessory proteins contribute differentially to the regulatory role of TrISW1 in achieving distinct patterns of gene expression.

10.1128/mbio.03456-21.2 TABLE S2 Orthologous ISW1 accessory subunits in Neurospora crassa, Trichoderma reesei, and Saccharomyces cerevisiae. Download Table S2, DOCX file, 0.02 MB. Copyright © 2022 Cao et al. 2022 Cao et al. https://creativecommons.org/licenses/by/4.0/ This content is distributed under the terms of the Creative Commons Attribution 4.0 International license.

10.1128/mbio.03456-21.7 FIG S5 Deletion or knockdown of ISWI and its accessory subunit genes hardly affected the vegetative growth of *T. reesei*. (A) Growth of the QM9414 and P*tcu1*-*Trisw1* strains on plates with a 1% glucose carbon source at 30°C for 3 days with or without copper. (B) Biomass accumulation of QM9414 and P*tcu1*-*Trisw1* in MA liquid culture with 1% glucose with copper. (C) Growth of QM9414, Δ*Triaf-1*, P*tcu1*-*Trioc4*^KD^, P*tcu1*-*Tracf-1*^KD^, and P*tcu1*-*Triaf-2*^KD^ strains on MA agar plates containing 1% glucose as the carbon source with or without copper. (D and E) Biomass accumulation of the above-listed strains in MA liquid culture with 1% glucose without (D) or with copper (E). Download FIG S5, TIF file, 2.9 MB. Copyright © 2022 Cao et al. 2022 Cao et al. https://creativecommons.org/licenses/by/4.0/ This content is distributed under the terms of the Creative Commons Attribution 4.0 International license.

10.1128/mbio.03456-21.8 FIG S6 Conditional turn-down/off of *Trisw1* expression compromised the induced cellulase gene expression in *T. reesei*. (A) Quantitative RT-PCR analyses of the relative transcription of *Trisw1* in the QM9414, P*tcu1*-*Trisw1*, and Δ*Trisw1* strains cultured with 1% Avicel for the indicated time points with or without copper. (B) Culture supernatant of the QM9414 and P*tcu*-*Trisw1* strains on 1% (wt/vol) Avicel supplied with copper was analyzed by SDS-PAGE and Coomassie brilliant blue staining. (C) The apparent color of the corresponding culture supernatant at 72 h for all detected strains. Download FIG S6, TIF file, 1.0 MB. Copyright © 2022 Cao et al. 2022 Cao et al. https://creativecommons.org/licenses/by/4.0/ This content is distributed under the terms of the Creative Commons Attribution 4.0 International license.

### TrISW1 acted in a differential relationship with YPR1 and XYR1 in Δ*Trisw1*.

**FIG 7:**
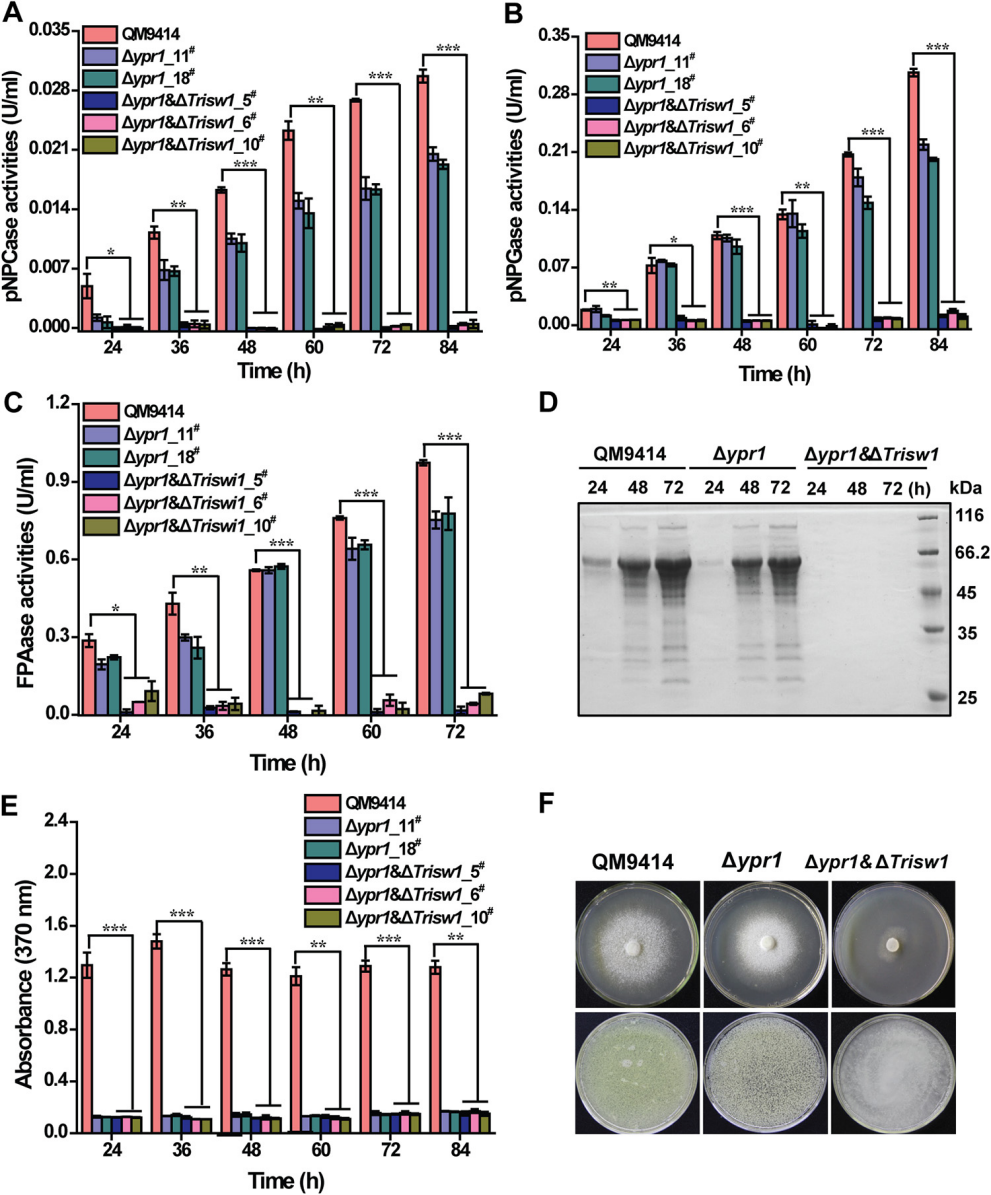
Simultaneous deletion of *ypr1* in Δ*Trisw1* eliminated sorbicillinoids production but failed to restore cellulase gene expression. (A to C) Extracellular *p*NPC (A), *p*NPG (B), and filter paper activities (FPA) (C) of the culture supernatant from QM9414, two independent transformants of Δ*ypr1*, and three independent transformants Δ*ypr1* Δ*Trisw1* cultured on 1% (wt/vol) Avicel for the indicated time periods. (D) Culture supernatant of the QM9414, Δ*ypr1*, and Δ*ypr1 *Δ*Trisw1* strains on 1% (wt/vol) Avicel was analyzed by SDS-PAGE and Coomassie brilliant blue staining. (E) The sorbicillinoid production of the QM9414, Δ*ypr1*, and Δ*ypr1 *Δ*Trisw1* strains cultured on 1% (wt/vol) Avicel was determined by measuring the absorbance at 370 nm at the indicated time points. (F) Growth of QM9414, Δ*ypr1*, and Δ*ypr1* Δ*Trisw1* on MA agar plates containing 1% glucose (upper panel) and the apparent color development of the corresponding culture supernatant at 84 h for the indicated strains (lower panel). Significant differences (*t* test *, *P* < 0.05; **, *P* < 0.01; ***, *P* < 0.001) were observed for extracellular activities and sorbicillinoid production between QM9414 and Δ*ypr1* Δ*Trisw1* for the indicated time points.

Considering that the transcription of *ypr1* coding for the key transcriptional activator of the *sor* cluster was significantly elevated without TrISW1, we wondered whether the enhanced production of the yellow pigment in Δ*Trisw1* resulted from *ypr1* deregulation. Simultaneous deletion of *ypr1* or *sor1* in Δ*Trisw1* was carried out to obtain the Δ*ypr1* Δ*Trisw1* and Δ*sor1* Δ*Trisw1* strains, respectively. Similar to Δ*sor1* Δ*Trisw1*, production of the yellow pigment was almost abolished in Δ*ypr1* Δ*Trisw1* (Fig. 7 and Fig. S7), indicating that the observed enhanced sorbicillinoid production resultant from *Trisw1* deletion necessitates YPR1, the deregulation of which may contribute to the elevated expression of the *sor* cluster. It has been shown that hyperproduction of the yellow pigment severely compromised extracellular cellobiohydrolase production (36). However, analysis of the extracellular hydrolytic activities of the two double deletion mutants revealed that elimination of the pigment did not ameliorate the defective cellulase induction (Fig. 7 and Fig. S7), indicating that the observed defect in cellulase gene expression in the absence of TrISW1 is not due to the enhanced formation of the yellow pigment.

10.1128/mbio.03456-21.9 FIG S7 Simultaneous deletion of *sor1* and *Trisw1* abolished both sorbicillinoid and cellulase production. (A to C) Extracellular *p*NPC (A), *p*NPG (B), and filter paper activities (FPA) (C) of the culture supernatant from the parental strain QM9414 and two independent transformants of Δ*sor1* and Δ*sor1* Δ*Trisw1* cultured on 1% (wt/vol) Avicel for the indicated time periods. (D) Culture supernatant of QM9414, Δ*sor1*, and Δ*sor1* Δ*Trisw1* on 1% (wt/vol) Avicel was analyzed by SDS-PAGE and Coomassie brilliant blue staining. (E) The sorbicillinoid production of QM9414, Δ*sor1*, and Δ*sor1* Δ*Trisw1* cultured in 1% (wt/vol) Avicel was assayed by measuring the absorbance at 370 nm at the indicated time points. (F) Growth of QM9414, Δ*sor1*, and Δ*sor1* Δ*Trisw1* on MA agar plates containing 1% glucose as the carbon source (upper panel) and the apparent color development of the corresponding culture supernatant at 84 h for the indicated strains (lower panel). Significant differences (*t*-test *, *P < *0.05; **, *P < *0.01; ***, *P < *0.001) were detected for extracellular activities and sorbicillinoid production between QM9414 and Δ*sor1* Δ*Trisw1* for the indicated time points. Download FIG S7, TIF file, 1.9 MB. Copyright © 2022 Cao et al. 2022 Cao et al. https://creativecommons.org/licenses/by/4.0/ This content is distributed under the terms of the Creative Commons Attribution 4.0 International license.

**FIG 8:**
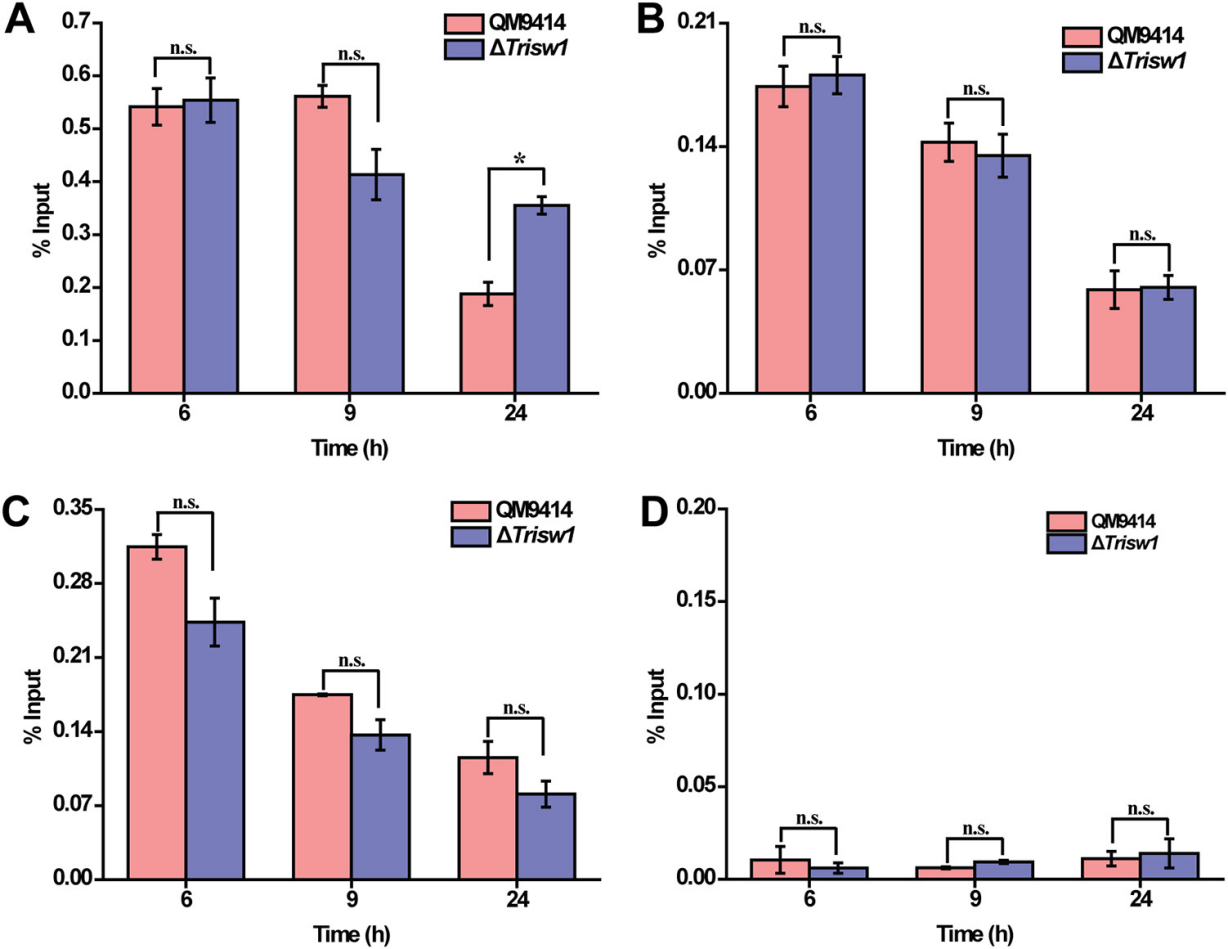
*Trisw1* deletion hardly has any effect on XYR1 occupancy on cellulase gene promoters. (A to D) ChIP analyses of XYR1 occupancy on *cbh1* (A), *cbh2* (B), *eg1* (C), and *actin* (D) promoters in the Δ*Trisw1* and QM9414 strains after induction for 6 h, 9 h, and 24 h on 1% (wt/vol) Avicel. Anti-XYR1 antibody was used to immunoprecipitate XYR1 bound to all detected cellulase gene promoters. No significant difference (*t* test *P* > 0.05 [n.s.]) was observed for XYR1 occupancy on cellulase gene or *actin* promoters between QM9414 and Δ*Trisw1* for the indicated time points.

Given that *xyr1* transcripts were otherwise severely compromised with *Trisw1* deletion, we wondered how *Trisw1* deletion affected XYR1 binding to cellulase gene promoters. Chromatin immunoprecipitation (ChIP)-qPCR analyses revealed that XYR1 occupancy on all the relevant cellulase gene promoters in Δ*Trisw1* was almost the same as that in QM9414 (Fig. 8). Taken together, these results suggest that TrISW1 is dispensable for XYR1 recruitment to cellulase gene promoters. TrISW1 may otherwise act downstream of XYR1 to contribute to changing the chromatin status of cellulase gene promoters to activate their transcription.

### TrISW1 is recruited to cellulase and *sor* gene promoters but exerts opposing effects on nucleosome positioning.

**FIG 9:**
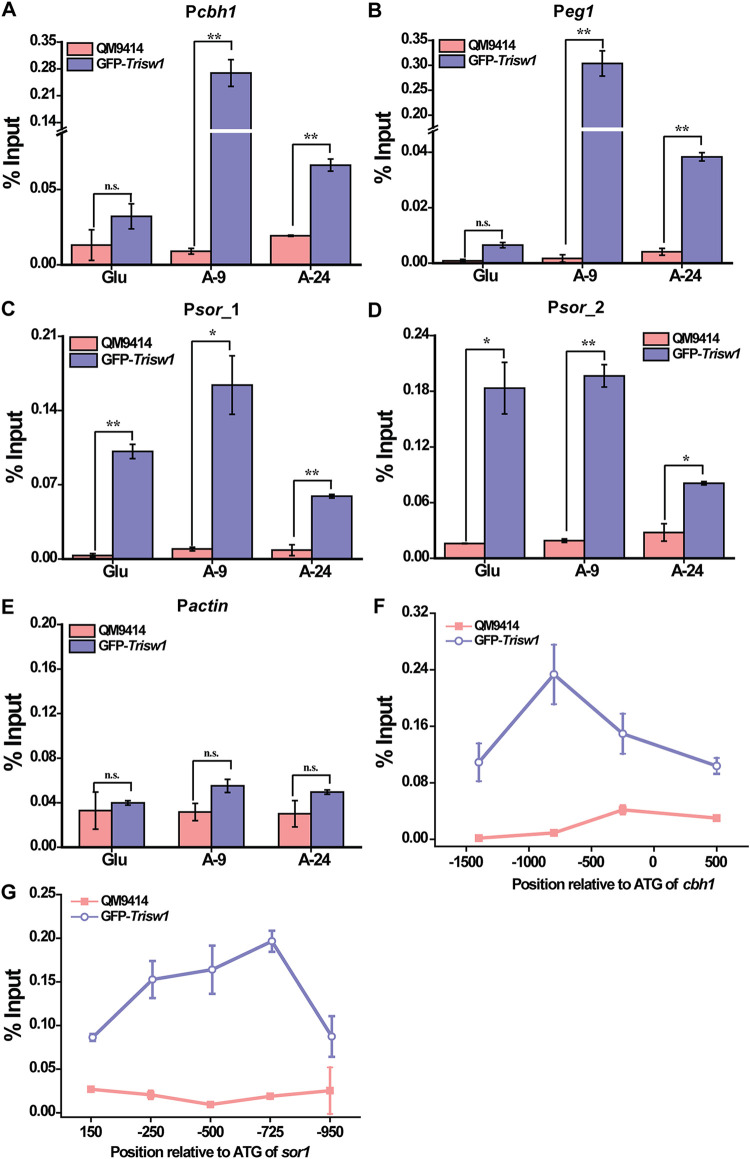
TrISW1 was recruited to cellulase and *sor* gene promoters. (A to E) ChIP analyses of TrISW1 occupancy on the *cbh1* (A), *eg1* (B), and *sor* (C-D) gene promoters, as well as the *actin* promoter (E) in the GFP-*Trisw1* and QM9414 strains cultured on 1% (wt/vol) glucose for 9 h (Glu) and on 1% (wt/vol) Avicel for 9 h (A-9) and 24 h (A-24). (F and G) ChIP analyses to provide an overview of TrISW1 occupancy over the *cbh1* (F) and *sor1* (G) promoters after Avicel induction for 9 h. The analyzed regions include *cbh1*-p1 (–1286 to −1427), *cbh1*-p2 (–664 to −905), *cbh1*-p3 (–179 to −355), and *cbh1*-ORF (418 to 603). For the *sor* promoter, the *sor1* ATG was set up as −1, and the analyzed regions include *sor1*-ORF (36 to 199), P*sor*_1 (−53 to −252), P*sor*_2 (−514 to −723), and *sor2*-ORF (−840 to −940). The numbers within brackets are the nucleotide position relative to the start codon ATG. Anti-GFP antibody was used to immunoprecipitate TrISW1 bound to all detected promoters. Significant differences (*t* test *, *P* < 0.05; **, *P* < 0.01) were observed for TrISW1 occupancy on *cbh1* and *eg1* promoters under Avicel induction for 9 h and 24 h, while no significant difference (*t* test *P* > 0.05 [n.s.]) was observed under glucose conditions. Significant differences (*t* test *, *P* < 0.05; **, *P* < 0.01) were observed for TrISW1 occupancy on *sor* promoters under both glucose- and Avicel-cultured conditions. No significant difference (*t* test *P* > 0.05 [n.s.]) was observed for TrISW1 occupancy on the *actin* promoter regardless of glucose or Avicel conditions.

To gain further insight into whether TrISW1 directly participates in the transcriptional regulation of cellulase and *sor* genes, chromatin immunoprecipitation (ChIP) was performed in QM9414 expressing a GFP-tagged TrISW1 under the control of P*tcu1*. As shown in Fig. 9A and B, a significant enrichment of TrISW1 was observed on promoter regions of the main cellulase-encoding genes (*cbh1* and *eg1*) only upon cellulose induction. Similarly, relatively higher TrISW1 recruitment was found on *sor* promoters regardless of the carbon source used (Fig. 9C and D). As expected, no significant enrichment of TrISW1 was detected on the *actin* promoter (Fig. 9E). An overview of TrISW1 occupancy over the whole *cbh1* promoter plus adjacent coding sequences further revealed that TrISW1 was significantly more enriched on a promoter region from −1,000 to −800 bp upstream of the start codon. Similarly, the enrichment signals for TrISW1 were increasingly higher at regions further upstream of than those downstream of ATG (Fig. 9F and G). Together, these data suggest that TrISW1 is directly involved in regulating cellulase and sorbicillinoid biosynthetic gene expression in *T. reesei*.

**FIG 10:**
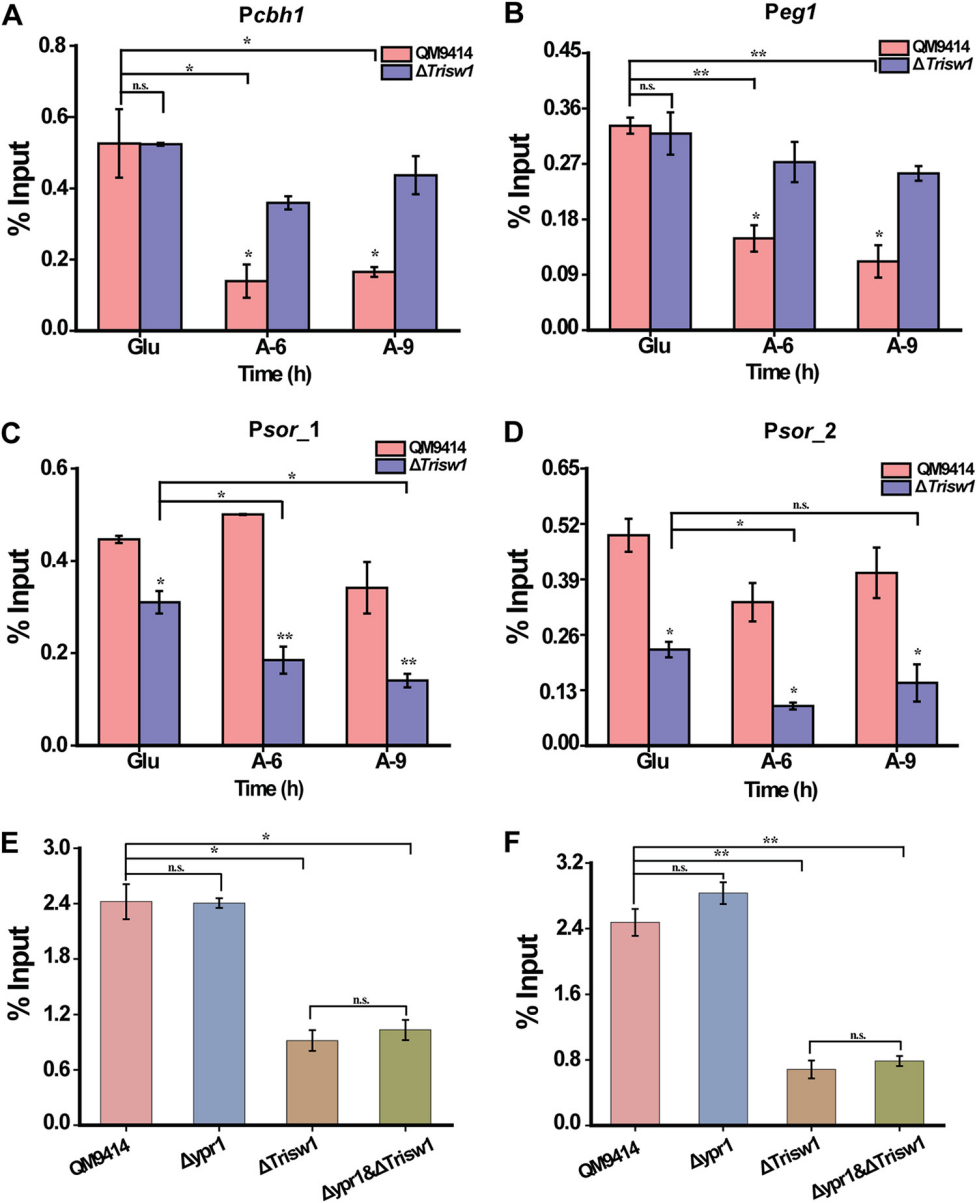
The effect of *Trisw1* deletion on the association of histone H4 with the *cbh1*, *eg1*, and *sor* gene promoters upon cellulose induction. ChIP assays were performed on chromatin isolated at different time periods after cellulose induction. (A and B) Histone H4 occupancy at the *cbh1* (A) and *eg1* (B) promoters in QM9414 upon cellulose induction, whereas the disassociation was impaired in the deletion strain. Significant differences (*t* test *, *P* < 0.05; **, *P* < 0.01) were detected for H4 association at the *cbh1* and *eg1* promoters in QM9414 between glucose and Avicel conditions as well as between QM9414 and Δ*Trisw1* upon Avicel induction. No significant difference (*t* test *P* > 0.05 [n.s.]) was observed for H4 association between QM9414 and Δ*Trisw1* on glucose cultured for 9 h (Glu). (C and D) Histone H4 disassociation was facilitated at the *sor* promoter in the *Trisw1* deletion strain. Significant differences (*t* test *, *P* < 0.05; **, *P* < 0.01) were detected for H4 association at the *sor* promoter between QM9414 and Δ*Trisw1* both under glucose and Avicel cultured conditions. (E and F) ChIP analyses of histone H4 occupancy at *sor* promoters in QM9414, Δ*ypr1*, Δ*Trisw1*, and Δ*ypr1* Δ*Trisw1* mutant strains on glucose for 9 h. Significant differences (*t* test *, *P* < 0.05; **, *P < *0.01) were detected for H4 association at *sor* promoters between QM9414 and Δ*Trisw1* as well as between QM9414 and Δ*ypr1* Δ*Trisw1*. No significant differences (*t* test, *P* > 0.05 [n.s.]) were observed for H4 association between QM9414 and Δ*ypr1* or between Δ*Trisw1* and Δ*ypr1* Δ*Trisw1* strains.

To test how TrISW1 recruitment is associated with chromatin status change in relevant gene promoters, loss of nucleosome components from the indicated promoter regions was tracked by ChIP to assay the loss of histone H4 (Fig. 10A to D). As reported earlier (33), a gradual but significant loss of histone H4 on cellulase gene promoters was observed with cellulose induction in QM9414 compared to noninducing conditions. However, H4 occupancy appeared to be continuously present in Δ*Trisw1* even with induction. In sharp contrast with cellulase genes, H4 occupancy at the *sor* promoter did not change significantly with cellulose induction in QM9414, but H4 disassociation had already occurred on glucose, and further loss of H4 was observed on cellulose in the deletion strain. To exclude the possibility that the observed loss of H4 is an indirect consequence of transcription, H4 occupancy at the *sor* promoter was determined in the Δ*ypr1* and Δ*ypr1* Δ*Trisw1* strains, where no *sor* gene transcription occurred (Fig. 10E and F) While deletion of *ypr1* alone had hardly any effect on H4 occupancy at the *sor* promoter compared to the control strain, the simultaneous absence of *ypr1* in Δ*Trisw1* did not prevent the significant loss of H4. In all, these data suggest that TrISW1 is differentially involved in remodeling nucleosomes positioned in cellulase and *sor* gene promoters to exert opposing effects on their expression.

## DISCUSSION

The filamentous fungus *T. reesei* is well known for its outstanding capacity of hyper-producing cellulase cocktail. Meanwhile, *T. reesei* can also produce a large amount of yellow pigment identified as a mixture of secondary metabolites with various bioactivities during cultivation (34, 35). Whereas it has been shown that hyper-production of sorbicillinoids is correlated with the dramatically reduced cellulase gene expression (36, 40), the involved regulatory mechanism that guarantees the subtle balance between cellulolytic response and sorbicillinoid biosynthesis is not clear in *T. reesei*. In this study, we found that TrISW1, the ATPase subunit of the ISWI complex, differentially participated in regulating the expression of cellulase genes and the gene cluster responsible for the yellow pigment biosynthesis in *T. reesei*. Deletion of *Trisw1* abolished cellulase gene expression but significantly enhanced the transcription of sorbicillinoid biosynthesis genes in *T. reesei*. These results indicate that TrISW1 plays a dual regulatory role in achieving a subtle balance between two physiological processes in *T. reesei*. On one hand, TrISW1 maintains a relatively low level of the major secondary metabolite biosynthesis by setting a brake on the expression of the relevant cluster genes. On the other hand, it contributes to the rapid cellulolytic response by facilitating the formation of an open chromatin configuration to ensure an efficient cellulase gene transcription initiation.

The transcriptional activation of cellulase genes in *T. reesei* is highly responsive to extracellular stimuli and therefore tightly controlled by a suite of transcription factors. Among them, XYR1 is identified as the master transcriptional activator that plays a dominant role in the induced expression of almost all cellulase and hemicellulase genes. In addition, several lines of evidence support the role of dynamic changes in the chromatin status in regulating transcription from cellulase gene promoters in *T. reesei* (31, 32, 41, 42). Nonetheless, the defective cellulase gene expression resultant from the loss of another chromatin remodeler TrSWI/SNF can be rescued by overexpression of XYR1 (our unpublished data), indicating that there may exist another chromatin remodeling complex participating in XYR1-mediated transcriptional activation. Our observation that TrISW1 is recruited to cellulase gene promoters upon cellulase induction implicates a direct involvement of TrISWI in the induced cellulase gene transcription. Indeed, loss of nucleosome components from the indicated promoter regions with induction was compromised in the absence of TrISW1. Although we do not have any evidence regarding how TrISW1 might be targeted to specific promoters, a possible candidate is XYR1, which has been shown to directly interact with TrSNF12 to recruit TrSWI/SNF (33). However, efforts to detect the direct interaction between XYR1 and TrISW1 were tried without success (data not shown). The possibility also exits that a yet-to-be-identified factor may act together with or independently of XYR1 to recruit TrISW1 upon cellulase induction. Moreover, considering that both SWI/SNF and ISWI are observed to be recruited to cellulase gene promoters in response to induction, an intriguing scenario is that these two complexes may act synergistically to aid in establishing a more open chromatin status that allows for more efficient binding of other transactivators as well as the transcription machinery, thus facilitating the initiation of cellulase gene transcription.

Contrary to cellulase gene expression, the transcription of the sorbicillinoid biosynthesis gene cluster appeared to be upregulated without TrISW1. Two transcription factors (YPR1 and YPR2) that are encoded in the same gene cluster have been shown to regulate the expression of *sor* genes (35). Our observations that *Trisw1* deletion led to an elevated transcription of *ypr1* and that simultaneous deletion of *ypr1* in Δ*Trisw1* eliminated the constitutive hyper-production of sorbicillinoids indicate that YPR1 represents a regulatory target of TrISW1. Similar to the case of cellulase genes, one reasonable possibility is that ISWI may be recruited by YPR2 to the *ypr1* promoter but instead exerts an inhibitory effect on its transcription. This assumption is consistent with the fact that the absence of YPR2 also results in the depression of YPR1, thus causing a highly activated expression of the *sor* genes and a hyper-production of sorbicillinoids (35). The possibility cannot be excluded, though, that TrISW1 directly participates in regulating the *sor* genes due to the observed enrichment of this factor on the relevant biosynthetic genes.

It has been reported that distinct ISWI complexes comprising multiple accessory subunits exist in other organisms, and the localization and catalytic activity of these ISWI complexes are subject to regulation by the associated subunits (12–18). Consistently, three distinct ISW-containing complexes with five discrete accessory subunits have been recently identified in *Neurospora* (18). Regardless of this, these distinct ISW complexes seem to perform an apparently overlapping role to regulate chromatin structure and gene repression at polycomb repressive complex 2 (PRC2) target domains. Contrary to the observations in *Neurospora*, we found that discrete homologous accessory subunits play differential roles in regulating the cellulase and *sor* gene expression. While the *Tracf-1* deletion mutant resembled Δ*Trisw1*, two other subunits (TrIAF-1 and TrIOC4) are specifically involved in the regulation of cellulase genes but not the *sor* gene expression. The requirement of TrIAF-1 and TrIOC4 for cellulase gene regulation implicates a role of these accessory subunits in specifically targeting the ISW1 complex to cellulase genes. In this respect, possible interactions between these subunits and known or hitherto uncharacterized transcriptional factors might exist to ensure the specific recruitment. On the other hand, the fact that TrACF-1 recapitulates TrISW1 function, rather, indicates that this subunit appears to be required for either the activity or the integrity of distinct ISW complexes. Once recruited, distinctly localized ISWI may help to create either a repressive or relatively open chromatin microenvironment that prevents or facilitates transcription (12–14, 27, 28, 43, 44). Whatever the case, the mechanism of TrISW1 recruitment as well as that of the formation of apparently different chromatin status with opposing effects on gene expression await further study.

Like all other organisms, fungi constantly face the challenge to outcompete other species to ensure efficient colonization in their natural habitat. They thus manage to either achieve fast growth on limited-nutrient resources or produce a versatile array of secondary metabolites to fight against competitors. Adoption of these two strategies is usually subtly controlled to balance the assignment of cellular resources. As a saprophytic fungus, *T. reesei* can rapidly synthesize a large amount of cellulases to hydrolyze the recalcitrant cellulose into fermentable sugar. On the other hand, it can produce secondary metabolites, including sorbicillinoids, that possess bioactive properties such as cytotoxic activities, antimicrobial activities, and antioxidant activities (34, 35, 45). Indeed, it has been reported that a *T. reesei* recombinant strain capable of hyper-producing yellow pigments can significantly inhibit the growth of plant-pathogenic fungi and promote its competition with pathogenic fungi (46). However, since both processes are expected to be extensively resource- and energy-expensive, it is instrumental for *T. reesei* cells to achieve a balance in allocating cellular resources to biosynthesize these two kinds of products when in need to guarantee a rapid growth and successful colonization. Nevertheless, *T. reesei* seems to produce more yellow pigments cultured on glucose than on cellulose (40). Our results thus point to a dual regulatory role of a chromatin remodeler in fine-tuning the occurrence of these two physiological processes. On one hand, TrISW1 acts as a repressor to set a brake on the sorbicillinoid biosynthetic gene transcription to maintain its relatively low-level expression. With cultivation, the inhibition exerted by TrISW1 is somehow released to allow the gradually accumulated sorbicillinoid production (36). On the other hand, TrISW1 is recruited to cellulase gene promoters on cellulase induction to facilitate the formation of a potentially more open chromatin environment, which contributes to the successful initiation of cellulase gene expression. The data thus provide a novel insight into how *T. reesei* takes advantage of a chromatin remodeler to exquisitely balance two different adaptive strategies to ensure an efficient allocation of cellular resources.

## MATERIALS AND METHODS

### Strains and cultivation conditions.

Escherichia coli DH5α cells were used for plasmid construction, which were cultured in lysogeny broth with a rotary shaker (200 rpm) at 37°C.

*T. reesei* QM9414 (ATCC 26921) and QM9414Δ*pyr4*, in which the uridine trophic marker gene was deleted in QM9414 (47), were used throughout this work as control and parental strains, respectively. All *T. reesei* strains were maintained on malt extract agar. For the transcription and (hemi)cellulase production analyses, *T. reesei* strains were pregrown in 1-L Erlenmeyer flasks on a rotary shaker (200 rpm) at 30°C in 250 mL Mandels-Andreotti (MA) medium with 1% (vol/vol) glycerol as the carbon source for 48 h as previously described (48). Mycelia were harvested by filtration and washed twice with medium without a carbon source. An equal wet weight (4 g) of mycelia was then transferred to fresh medium without peptone containing 1% (wt/vol) Avicel or other carbon sources as indicated, and incubation was continued for the indicated time periods.

### Plasmid and strain construction.

To delete *Trisw1* (Tr_57608) and *Trino80* (Tr_50539), DNA fragments corresponding to approximately 2.2 kb of *Trisw1* or *Trino80* upstream noncoding regions were amplified from QM9414 genomic DNA and inserted into the HindIII and PmeI sites of the pUC19-*pyr4* plasmid (49) to obtain pUC19-*pyr4_Trisw1*up or pUC19-*pyr4_Trino80*up. Similarly, approximately 2.0 kb of *Trisw1* or 2.2 kb of *Trino80* downstream noncoding regions was amplified and ligated into pUC19-*pyr4_Trisw1*up or pUC19-*pyr4_Trino80*up after digestion with BamHI/EcoRI to generate pUC19-*pyr4_Trisw1* or pUC19-*pyr4_Trino80*. To delete *Trchd1* (Tr_58928) or *Triaf-1* (Tr_43919), DNA fragments corresponding to approximately 2.0 kb of *Trchd1 or Triaf-1* up- and 2.2 kb of *Trchd1 or Triaf-1* downstream noncoding regions were amplified from QM9414 genomic DNA and inserted into the HindIII/PmeI and BamHI/EcoRI sites of the pUC19-*pyr4* plasmid to obtain pUC19-*pyr4_Trchd1* or pUC19-*pyr4_Triaf-1.* The pUC19-*pyr4_Trisw1* and pUC19-*pyr4_Triaf-1* were used to transform *T. reesei* QM9414Δ*pyr4* after linearization with EcoRI to obtain the Δ*Trisw1* and Δ*Triaf-1*, respectively. pUC19-*pyr4_Trino80* and pUC19-*pyr4_Trchd1* plasmids were linearized with HindIII and transformed into *T. reesei* QM9414Δ*pyr4* to generate Δ*Trino80* or Δ*Trchd1*, respectively.

To knock down *Trioc4*, *Tracf1*, or *Triaf-2* expression using an RNA interference approach, a 320-bp fragment of *Trioc4*, an 1.2-kb fragment of *Tracf1*, or a 1.1-kb fragment of *Triaf-2* within coding sequences was amplified with *T. reesei* genomic DNA as the template and ligated in a reversed manner into EcoRV/KpnI and SpeI/NotI sites of the pKD-*hph* plasmid (50) to obtain pKD-*hph*-*T Trioc4*, pKD-*hph*-*Tracf1*, and pKD-*hph*-*Triaf-2*, respectively. These plasmids were transformed into QM9414 to result in the P*tcu1*-*Trioc4*^KD^, P*tcu1*-*Triacf1*^KD^, or P*tcu1*-*Tracf-2*^KD^ strains where *Trioc4*, *Tracf1*, or *Triaf-2* expression was repressed without copper but remained unaffected when 20 mM copper was included.

To construct the P*tcu1*-based promoter replacement vector for *Trisw1*, the 1.9-kb flanking sequence upstream from the initiation codon ATG and a 3.5-kb fragment downstream from ATG of the *Trisw1* gene were amplified from genomic DNA of QM9414, digested with HindIII/AscI and NotI/SpeI, respectively, and ligated into the corresponding sites of the pMDP*tcu1*-*pry4* plasmid (51) sequentially to obtain pMDP*tcu1*-*Trisw1*. This plasmid was linearized with HindIII before being transformed into *T. reesei* QM9414Δ*pyr4* to obtain P*tcu1*-based promoter replacement strain P*tcu1*-*Trisw1*. The *Trisw1* coding sequence was amplified from QM9414 genome DNA, digested with NcoI/SpeI, and then ligated into pMDP-*gpd*-*hph* (33) to obtain the pMDP-*gpd*-*hph-Trisw1* plasmid. The TrISW1-K195R mutant was obtained by overlap-extension PCR (52, 53) and similarly inserted into the pMDP-*gpd*-*hph* plasmid to generate pMDP-*gpd*-*hph-Trisw1-*K195R. These plasmids were transformed into the P*tcu1*-*Trisw1* strain to obtain the Re*Trisw1* and Re*Trisw1-*K195R strains, respectively.

To determine the subcellular localization of TrISW1, the TrISW1 coding sequence was amplified from *T. reesei* cDNA and inserted into the NotI and SpeI sites in the P*tcu1*-EGFP-*hph* plasmid (51) to obtain P*tcu1*-EGFP-*Trisw1*. This plasmid was transformed into QM9414 to obtain the GFP-*Trisw1* strain. The same strategy was used for construction of the GFP-TrISW1-K195R strain, which is used for the determination of the localization of the TrISW1-K195R mutant.

In order to construct the Δ*ypr1* Δ*Trisw1* and Δ*sor1* Δ*Trisw1* strains, DNA fragments corresponding to approximately 1.8 kb and 2.2 kb of *ypr1* or *sor1* upstream noncoding regions were amplified from QM9414 genomic DNA and inserted into the HindIII and SalI sites of the pUC19-*hph* plasmid (49) to obtain pUC19-*hph-ypr1*up or pUC19-*hph-sor1*up. Approximately 2.0 kb of *ypr1* or 2.1 kb of *sor1* downstream noncoding regions were then ligated into pUC19-*hph-ypr1*up or pUC19-*hph-sor1*up after digestion with ApaI/EcoRI to generate pUC19-*hph-ypr1* or pUC19-*hph-sor1.* These two plasmids were individually transformed into QM9414Δ*pyr4* to generate Δ*ypr1* and Δ*sor1*, respectively. The pUC19-*pyr4-Trisw1* plasmid was finally transformed into the Δ*ypr1* or Δ*sor1* strain to generate Δ*ypr1* Δ*Trisw1* or Δ*sor1* Δ*Trisw1*.

*T. reesei* transformation was carried out essentially as previously described (49). The transformants were selected on minimal medium for either uridine prototroph or resistance to hygromycin (120 μg/mL). Anchored PCR was used to verify the correct integration events.

### Vegetable growth and conidiation assays.

To analyze *T. reesei* vegetative growth, strains were precultured on minimal medium agar plates for 2 days. A slice of agar with the same area of growing mycelia of the indicated strain (1 cm in diameter) was taken from the plate and inoculated on minimal medium agar plates containing different carbon sources (glucose, glycerol, cellobiose, or lactose, 1% [wt/vol]) at 30°C for 3 days or on malt extract agar plates to be incubated for 5 days. To determine *T. reesei* biomass accumulation in liquid MA medium with 1% (wt/vol) different carbon sources (glucose, glycerol, cellobiose, lactose, or Avicel), equal amounts of mycelia were inoculated, and mycelia collected at the indicated growth intervals were dried and weighed or broken for determining the intracellular protein content (54).

### Enzymatic activity and protein analysis.

Cellulolytic enzyme activity was determined as previously described (49, 55). Briefly, cellobiohydrolase and β-glucosidase activities were determined by measuring the amount of released *p*-nitrophenol using *p*-nitrophenyl-d-cellobioside (*p*NPC; Sigma) and *p*-nitrophenyl-β-d-glucopyranoside (*p*NPG; Sigma) as the substrates, respectively. The cellulase activity assays were performed in 200-μL reaction mixtures containing 50 μL of culture supernatant and 50 μL of the respective substrate plus 100 μL of 50 mM sodium acetate buffer (pH 4.8) and then incubated at 45°C for 30 min (49). One unit (U) of *p*NPCase activity is defined as the amount of enzyme releasing 1 μmol of *p*NP per minute. Xylanase activities were determined by measuring the amount of released xylose using xylan as the substrate. Briefly, a reaction mixture containing 60 μL of appropriately diluted culture supernatant and 60 μL of beechwood xylan (5 g/L) dissolved in 50 mM sodium acetate buffer (pH 4.8) was incubated at 50°C for 15 min. The reducing sugar released in the mixture was determined using dinitrosalicylic acid (DNS) method with xylose as the standard. The endo-glucanases and filter paper activities (FPA) were determined by measuring the released reducing sugar with carboxymethylcellulose sodium salt (CMC; Sigma) and filter paper as substrates, respectively. Determination of CMC hydrolytic activities was carried out at 50°C in a 100- μL reaction mixture containing 50 μL of appropriately diluted culture supernatant and 50 μL of 0.5% (wt/vol) CMC sodium in 50 mM sodium acetate buffer (pH 4.8). The FPA assay was performed at 50°C in a 200-μL reaction mixture including 50 μL of appropriately diluted culture supernatant and 150 μL of 50 mM sodium acetate buffer (pH 4.8) with Whatman no. 1 filter paper as the substrate. One unit (U) of CMCase or FPA was defined as the release of 1 μmol reducing sugar per minute under the test conditions. To control for the difference in growth rate, equal large amounts of precultured mycelia were transferred to Avicel medium to minimize the effect of growth differences with induction. An equal amount of culture supernatant was then loaded for enzymatic activity and SDS-PAGE analysis. An equal amount of culture supernatant was then used for enzymatic activity and protein analysis. SDS-PAGE was performed essentially as previously described (56).

### Quantitative RT-PCR.

Total RNA was extracted using TRIzol reagent (Vazyme, Nanjing, China) and purified using the TURBO DNA-free kit (Invitrogen, USA) to remove genomic DNA (gDNA) according to the manufacturer’s instructions. Reverse transcription was carried out using the PrimeScript RT reagent kit (Vazyme) according to the instructions. Quantitative PCR was performed on a LightCycler 480 II (Roche, Basel, Switzerland). Amplification reactions were performed using the SYBR green supermix (Vazyme) according to the manufacturer’s instructions. Data analysis was performed using the relative quantitation/comparative CT (ΔΔCT) method, and the results were normalized to an endogenous control (actin), with the expression level on glycerol as the reference sample (57). Two or three biological replicates were performed for each analysis, and the results and errors are the mean and the standard deviation (SD), respectively, from the replicates. Statistical analysis was performed using Student’s *t* test analysis.

### Chromatin immunoprecipitation (ChIP) analyses.

ChIP assays were performed according to a previously described protocol (58, 59). Briefly, the mycelia were fixed in minimal medium containing 1% formaldehyde at 30°C for 10 min with shaking before the cross-linking was quenched via the addition of 25 mL of 1.25 M glycine for 5 min. The mycelia were then collected, suspended in lysis buffer (50 mM HEPES, pH 7.5, 150 mM NaCl, 1 mM EDTA, 0.5% Triton X-100, 0.1% sodium deoxycholate, 0.1% SDS, 1 mM PMSF [phenylmethanesulfonyl fluoride], 1 μg/mL leupeptin, and 1 μg/mL pepstatin) with glass beads (0.45 mm). Chromatin DNA was further sonicated to obtain sheared DNA fragments with an average size of approximately 500 bp. Immunoprecipitation was conducted with the antibodies against XYR1 (59), H4 (Millipore, USA) (33), and GFP (Santa Cruz Biotechnology, USA). Quantitative PCR was performed on the precipitated chromatin DNAs using the same procedure as with qRT-PCR. Relative enrichment of the DNAs was calculated as a percentage of the input DNA. The corresponding promoter regions used for amplification in ChIP assays are shown in Fig. S8.

10.1128/mbio.03456-21.10 FIG S8 Schematic demonstration of cellulase and *sor* gene promoters and the location of primers used for ChIP-qPCR. The number below each short bar denotes the approximate position of the amplified promoter regions relative to the start codon ATG, which was set up as +1. Download FIG S8, TIF file, 0.5 MB. Copyright © 2022 Cao et al. 2022 Cao et al. https://creativecommons.org/licenses/by/4.0/ This content is distributed under the terms of the Creative Commons Attribution 4.0 International license.

### Resting cell-induced gene expression assay.

The resting cell-inducing system was performed as previously described (49). Briefly, strains were precultured in Mandels-Andreotti (MA) medium with 1% glycerol for 48 h. Mycelia were harvested by filtration and washed twice with medium without a carbon source, and then all mycelia were transferred to fresh medium with no carbon source and cultured at 30°C for 1 h on a rotary shaker (200 rpm) to deplete any intracellularly accumulated carbon and nitrogen sources. The mycelia were collected again, washed twice with 20 mM sodium citrate (pH 5.0), and equal wet weights (4 g) of mycelia were then transferred to 250 mL of 20 mM sodium citrate supplemented with 1% Avicel. The mycelia were collected at the indicated growth intervals for quantitative RT-PCR assays.

### Fluorescence microscopy.

To visualize GFP-*Trisw1*, recombinant strain spores were inoculated and germinated in MA medium containing either 1% (vol/vol) glucose for 16 h or 1% (wt/vol) Avicel for 24 h at 30°C. After incubation, germlings were fixed on the coverslips using methanol and then stained with 100 μg/mL of DAPI (4′,6-diamidino-2-phenylindole dihydrochloride) solution in 50% glycerol for 5 min. The fluorescence of GFP-*Trisw1* was detected with an Eclipse 80i fluorescence microscope (Nikon, Melville, NY, USA), and images were captured and processed with the NIS-ELEMENTSAR software. The same strategy was used for the fluorescence of the GFP-TrISW1-K195R strain.

### High-pressure liquid chromatography (HPLC)-MS.

To verify sorbicillinoid production, culture supernatant was first filtered with a G1 funnel. The same amount of ethyl acetate was then added and gently mixed before being allowed to rest for 30 min. The upper organic phase was collected and evaporated by rotary evaporator, and the dry matter was dissolved in 3 mL methanol and filtered using a 0.22-μm-aperture filter. Then a 10-μL sample was injected into a C_18_ column at a flow rate of 1 mL/min using H_2_O plus 0.1% formic acid (A) and acetonitrile plus 0.1% formic acid (B) as the mobile phase. The UV spectrum detection range is 300 to 500 nm, and the molecular weight collection range (*m/z*) is 50 to 1,500 (positive ion).

### Determination of the optical absorbance of *T. reesei* culture supernatant.

*T. reesei* conidia were inoculated into MA liquid medium containing 1% glycerol as the carbon source and precultured for 36 h. Equal amounts of filtered and washed mycelia were collected and transferred to MA medium containing 1% virous carbon source. After cultivation for the indicated periods, the culture supernatant of each strain was collected and subjected to determination of the optical absorbance at 370 nm using a microplate reader (BioTek).

### Statistical analysis.

Statistical analysis was performed using Student’s *t* test analysis. At least two to three biological replicates were performed for each analysis, and the results and errors are the mean and SD, respectively, of these replicates.
