# Supplementary material for: Dual Regulatory Role of Chromatin Remodeler ISW1 in Coordinating Cellulase and Secondary Metabolite Biosynthesis in Trichoderma reesei
Source: mBio. 2022 Feb 8;13(1):e03456-21. doi: 10.1128/mbio.03456-21 (PMC8822348; doi:10.1128/mbio.03456-21)
Supplement: TABLE S1 [file mbio.03456-21-st001.docx]

**Table S1. LC-MS results of the extracted culture broth of Δ*Trisw1* grown on galactose for 96 h**

| **compound** | **name** | **Formula** | **Acquired [M+H]^+^** | **RT (min)** |
| --- | --- | --- | --- | --- |
| 1 | Sorbicillinol | C_14_H_16_O_4_ | 249.1135 | 26.2 |
| 2 | Bisvertinolone | C_28_H_32_O_9_ | 513.2129 | 30.1 |
| 3 | Bisorbicillinol | C_28_H_32_O_8_ | 497.2168 | 26.2 |
| 4 | Sorbicillin | C_14_H_16_O_3_ | 233.1177 | 29.8 |
| 5 | unknown | C_14_H_16_O_9_ | 327.0778 | 30.7 |
| 6 | unknown | C_27_H_24_O_2_N_7_ | 479.2061 | 25.9 |
