# Supplementary material for: Dual Regulatory Role of Chromatin Remodeler ISW1 in Coordinating Cellulase and Secondary Metabolite Biosynthesis in Trichoderma reesei
Source: mBio. 2022 Feb 8;13(1):e03456-21. doi: 10.1128/mbio.03456-21 (PMC8822348; doi:10.1128/mbio.03456-21)
Supplement: TABLE S2 [file mbio.03456-21-st002.docx]

**Table S2 Orthologous ISW1 accessory subunits in *Neurospora crassa*, *Trichoderma reesei,* and *Saccharomyces cerevisiae***

| *N. crassa* orthologs | | | *T. reesei* orthologs | Yeast subunits | | |
| --- | --- | --- | --- | --- | --- | --- |
| NCU03875 (ISW, 56.77%^a^) | | Tr57608 (TrISW1, 55.80% ^a^, 84.39% ^b^) | | | ISW1/2 | |
| NCU00412 (IAF-1: 21.46% ^a^)  NCU02684 (NcIOC4, 23.40%^a^)  NCU00164 (ACF1: 27.20% ^a^)  NCU09388 (IAF-2)  NCU03073 (HSP-1: 29.02% ^a^)  NCU06623 (HSP-2: 23.47% ^a^) | Tr43919 (TrIAF-1:21.89%^a^, 58.53% ^b^)  Tr63747 (TrIOC4:27.05%^a^, 55.43% ^b^)  Tr61508 (TrACF1:26.27%^a^, 61.12% ^b^)  Tr104643 (TrIAF-2: 48.93% ^b^)  Tr108421(28.5% ^a^, 50.18%^b^)  Tr62979 (26.16% ^a^, 61.88%^b^) | | | | | IOC3  IOC4  ITC1  un  NP_010406.3  NP_009837.1 |

*Trichoderma reesei* homologs were retrieved using BlastP with the corresponding *Neurospora* *crassa* subunits as queries. *Saccharomyces* *cerevisiae* orthologous counterparts were listed for comparison. Although not reported by Kamei et al. (1), *Neurospora* *crassa* and *T. reesei* orthologs of the yeast IOC4 subunit with low sequence identity were identified. ^a^ Amino acid identity to the *S. cerevisiae* counterparts; ^b^ Amino acid identity between *T. reesei* and *N*. *crassa* orthologs. un. not identified

**Reference**

1. Kamei M, Ameri AJ, Ferraro AR, Bar-Peled Y, Zhao F, Ethridge CL, Lail K, Amirebrahimi M, Lipzen A, Ng V, Grigoriev IV, Schmitz RJ, Liu Y, Lewis ZA. 2021. IMITATION SWITCH is required for normal chromatin structure and gene repression in PRC2 target domains. Proc Natl Acad Sci U S A 118.
